# Direct Evidence for the Presence of Human Milk Oligosaccharides in the Circulation of Breastfed Infants

**DOI:** 10.1371/journal.pone.0101692

**Published:** 2014-07-07

**Authors:** Karen C. Goehring, Adam D. Kennedy, Pedro A. Prieto, Rachael H. Buck

**Affiliations:** 1 Department of Global Discovery Research, Abbott Nutrition, Columbus, Ohio, United States of America; 2 Metabolon, Inc., Durham, North Carolina, United States of America; Indiana University, United States of America

## Abstract

**Background:**

It has been hypothesized that human milk oligosaccharides (HMOs) confer systemic health benefits to breastfed infants; however, plausible mechanisms for some effects, such as systemic immunomodulation, require HMOs to access the bloodstream of the developing infant. While small concentrations of HMOs have been detected in the urine of breastfed infants there are no published studies of these oligosaccharides accessing the plasma compartment of breastfed infants. Here we determined the relative fractions of several ingested HMOs in infant urine and plasma. Plasma from formula-fed infants was used as a control.

**Methods:**

Using gas chromatography/mass spectrometry (GC/MS), liquid chromatography/mass spectrometry/tandem mass spectrometry (LC/MS/MS), and high performance liquid chromatography (HPLC), we analyzed the urine and plasma from 17 healthy formula-fed infants and 16 healthy breast-fed infants (and the milk from their mothers).

**Results:**

Multiple HMOs were detected in the urine and plasma of breastfed infants, but not in formula-fed infants. Levels of 2′-fucosyllactose (2′FL), 3FL and lacto-N-neotetraose (LNnT) in both plasma (r = 0.98, p<0.001; r = 0.75, p = 0.002; r = 0.71, p = 0.004) and urine (r = 0.81, p<0.001; r = 0.56, p = 0.026; NS) correlated significantly with concentrations in the corresponding breast milk. The relative fractions of HMOs were low, 0.1% of milk levels for plasma and 4% of milk levels for urine. Within the breastfed cohort, there were significant differences between secretor and nonsecretor groups in levels of several fucosylated HMOs.

**Conclusion:**

At least some ingested HMOs are absorbed intact into the circulation and excreted in the urine and their concentrations in these fluids correlate with levels of the corresponding mother's milk. While relative fractions of absorbed HMOs were low, these levels have been shown to have biological effects *in vitro*, and could explain some of the postulated benefits of human milk.

## Introduction

Human milk contains large amounts of soluble milk oligosaccharides, 5–23 g/L, representing the third largest solid component following lactose and lipids [1]. Milk oligosaccharides are present at significantly higher concentrations and in greater diversity in human milk than in bovine milk [2]. These glycans have typically been called human milk oligosaccharides (HMO or HMOs), even though many of them are also present in the milk of other animals. HMOs are composed of five monosaccharides: d–glucose (Glc), d–galactose (Gal), *N*–acetylglucosamine (GlcNAc), l–fucose (Fuc), and *N*–acetylneuraminic acid (Neu5Ac), with a lactose core at the reducing end. There have been over 200 HMOs identified in human milk thus far, with levels and patterns varying between individuals and over the course of lactation [1], [3]–[6]. About 20 structures, including 2′-fucosyllactose (2′FL), comprise the major portion of HMOs, with the remaining glycans representing only a small fraction [6]. Fucosylation of HMOs is genetically determined and reflective of Secretor (*Se*) and Lewis (*Le*) histo-blood group antigen status. The *Se* locus encodes for α1,2 fucosyltransferase (FUT2), while the *Le* locus encodes α1,3/4 fucosyltransferase (FUT3). HMO structures containing (α1,2)-linked fucose, such as 2′FL, require the activity of α1,2 fucosyltransferase, and are therefore absent from the milk of women homozygous for mutations in the FUT2 gene (historically known as ‘nonsecretors’, *Se-*). In milk from ‘secretor’ (*Se*+) women, 2′FL is one of the major oligosaccharides. Secretors make up the major part of European and American populations, about 80% of the population [7], [8]. In a recent study profiling the HMO content of breast milk, Totten et al. proposed a method for determining secretor status based exclusively on the relative quantitation of milk oligosaccharides. They determined a 2′FL/3FL abundance ratio of >6.5 to be a specific marker for describing an individual as a secretor, and were able to correctly identify 42 out of 44 secretors. The two samples that were assigned Le(a−b+) (secretor) status by serological tests were found to produce the markers in minute amounts comparable to that of nonsecretors [9]. Erney et al. [10] analyzed hundreds of milk samples for HMO content, making special effort in detecting and quantifying 2′FL. They determined that western blots of proteins from milk samples that contained even minute quantities of 2′FL were detected by *Ulex Europeaus* agglutinin I, thus providing a different criterion to assign secretor status to milk samples in the absence of serological determinations. Examples of these western blots and their ability to predict secretor status are shown in Prieto [11].

Exclusive breastfeeding has been associated with a reduced risk of infection in infants [12]–[16]. The protective properties of human milk have historically been attributed to antibodies and other bioactive molecules, such as nucleotides and cytokines [17], [18]; however, recent evidence suggests that milk oligosaccharides, may also play a significant role. In a recent study, LNFP-II (lacto-N-fucopentaose II), a glycan present in human milk, was measured as a representative of total levels of HMOs present. The level of LNFP-II in maternal milk at 2 weeks postpartum (8 mg/L) was associated with fewer respiratory and enteric problems in infants by 6 and 12 weeks of age [19].

The underlying mechanisms of action for these beneficial effects are not fully understood and likely involve multiple tactics: (1) *in vitro* data suggest that HMOs act as prebiotics, selectively promoting the growth of beneficial bacteria, while suppressing the growth of pathogens [20]–[24]; (2) HMOs coat the infant's mucosal surfaces and may act as soluble receptor analogues, inhibiting the attachment of pathogenic microorganisms [21], [25], [26]; (3) HMOs interact with specific glycan binding proteins differentially expressed by nearly all epithelial and immune cells, thus may directly modulate immune system responses and development, and gut maturation [21], [27]–[29]; (4) HMOs may also provide the infant with essential factors for brain and cognitive development [21], [26]. Researchers have demonstrated that HMOs can be translocated or actively transported through cell monolayers and that ^13^C-labeled glycans are found in the urine of breastfed infants, thus implying passage through the plasma compartment [30], [31].

The purpose of this study was to explore the fate of milk glycans once they are consumed by the infant, and to investigate the influence of breast milk with its high content of HMOs compared to that of cow's-milk based infant formula, containing low levels of only a few small oligosaccharides. We also looked at 2′FL and 6′SL, as representatives of major fucosylated and acidic glycans present in human milk, to determine the relationship between levels found in the mother's milk and those found in the infant's urine and plasma.

## Methods

### Ethics Statement

The study was carried out according to the Declaration of Helsinki and followed ICH-GCP guidelines. Institutional review board approval (Copernicus Group IRB) was obtained at each participating site, and written informed consent was obtained from all mothers/legal guardians prior to participation in the study.

### Study Design

**Figure 1:**
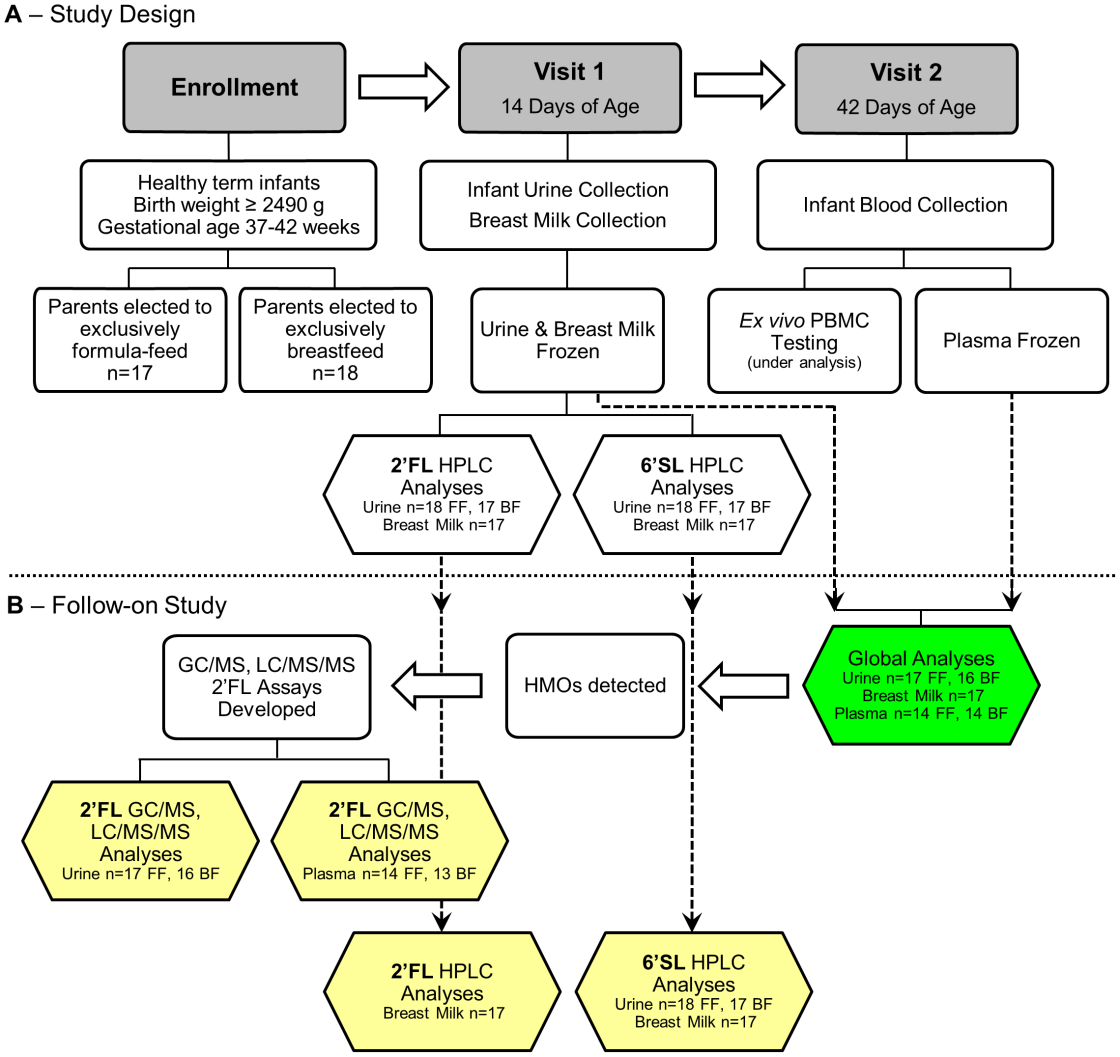
Schematic of Study Design. Targeted analyses generating “Absolute Quantitation” are shown in yellow. Global analyses generating “Relative Quantitation” are shown in green. Dotted arrows indicate samples and analyses from Study A used in our Follow-on Study B.

This study utilized samples collected from a pilot study (Figure 1A), based on Stepans et al. [19], that compared the levels of 2′FL and 6′SL in mother's milk at day 14 postpartum with an *ex vivo* innate immune response in infant PBMCs collected at day 42 postpartum (analysis ongoing). Infant urine was also collected on day 14 to look at the relationship between breast milk and urine levels of 2′FL and 6′SL.

Using surplus frozen milk, urine and plasma samples (Figure 1B), we performed “Global, Untargeted Analyses” of the samples. Upon the detection of multiple HMOs in the samples, “Targeted Analyses” for 2′FL were developed to provide the “Absolute Quantitation” of 2′FL in the urine and plasma samples. 2′FL was selected because it is the most abundant HMO in human milk. We chose to use the existing HPLC data generated for the original study for the “Absolute Quantitation” of 2′FL and 6′SL in breast milk, and 6′SL in urine.

### Sample Donors and Sample Collection

Samples were obtained from two groups (Figure 1A), exclusively breastfeeding infant-mother dyads and infants receiving a commercial infant formula fortified with dietary galactooligosaccharides [Similac Advance Powder, Abbott Laboratories, Columbus, OH]. All infants were healthy, singletons from a full term birth with a gestational age of 37–42 weeks, whose parents had elected to either exclusively breastfeed or formula-feed. Plasma and urine were collected from infants, and human milk was collected from those mothers who had elected to breastfeed.

A morning, mid-feed milk sample was obtained from one breast via a breast milk pump, 14 days postpartum. Around 3–5 mL of milk were dispensed into sterile plastic tubes, frozen immediately, and stored at −20°C until analysis.

A non-fecal contaminated infant urine sample was collected from an absorbent pad (Uricol - The Newcastle Urine Collector) placed in the diaper, 14 days postpartum, on the day of milk sampling. Urine was retrieved from the pad within 60 minutes of voiding, by pressing the tip of a sterile syringe into the pad and withdrawing the plunger. 1–3 mL of urine were then transferred into sterile plastic tubes, frozen immediately, and stored at −20°C until analysis.

2–3 ml of non-fasting blood were collected from the infant's antecubital vein into sodium heparin vacutainer tubes at 42 days postpartum. Blood samples were shipped at ambient temperature to the lab, and received within 24 hours of collection. Plasma was obtained by standard centrifugation procedure, dispensed into small plastic tubes, and stored at −80°C until analysis.

### Global Metabolomic Analysis

For relative quantitation, biochemical profiling was performed as described previously for the analysis of plasma and urine [32], [33]. Briefly, samples of human milk, and plasma and urine from breastfed (BF) and formula-fed (FF) infants were extracted and split into equal parts for analysis on the GC/MS and LC/MS/MS platforms. This method focused on those biochemicals between 50 and 1,000 Da in molecular weight [34]. Many of the major HMOs fall within that description. We performed chromatographic separation, followed by full-scan mass spectroscopy, to record and identify all detectable ions formed after fragmenting the molecules in each of the samples. Software was used to match ions to an in-house library of standards for metabolite identification and for metabolite quantitation by peak area integration [35]. We identified metabolites with known chemical structure by matching the ions' chromatographic retention index, nominal mass, and mass spectral fragmentation signatures with reference library entries created from authentic standards for each metabolite under the identical analytical procedure as the experimental samples.

### Targeted Analysis

For absolute quantitation of 2′FL in urine and plasma, we developed assays using isotopically labeled standards and developed standard curves with these stable isotope dilution assays. The standard curve for plasma was 0.05 to 5 mg/L and for urine was 0.05 to 50 mg/L. Samples were spiked with an internal standard and subjected to protein precipitation with methanol. Following centrifugation, aliquots of clear supernatant were injected onto an Agilent 1290/AB Sciex QTrap 5500 LC MS/MS system equipped with a BEH Amide normal phase UHPLC column. We performed quantitation based on the area ratios of 2′FL and internal standard peaks using a weighted linear least squares regression analysis generated from freshly prepared calibration standards. Relative recoveries of 2′FL were 101% in plasma and 84% in urine. QC RSD values were very low (4.1% for plasma and 4.2% for urine). Additionally, precision for inter run RSD for urine was 2.3 to 5.0% and 2.5 to 7.2% for plasma. Comparison of the targeted, absolute quantitative data to the global platform relative quantitation showed ∼90% correlation in each matrix.

Content of 2′FL and 6′SL in human milk and 6′SL in urine was measured using established high-performance liquid chromatography (HPLC) procedures [36], kindly performed at the University of Illinois.

### Data Normalization and Statistical Analyses

For the global metabolomic analyses, “Relative Quantitation”, the raw area counts for each oligosaccharide in each sample matrix were normalized to correct for variation resulting from instrument inter-day tuning differences [34]. Missing values were assumed to result from areas falling below the limits of detection for that oligosaccharide with the instrumentation used and were imputed with the observed minimum for that sample matrix. For the convenience of data visualization, the raw peak areas for each oligosaccharide were then normalized by dividing each sample value by the median value for the specific oligosaccharide and sample matrix, giving a relative “scaled intensity”. This correctly preserves the variation between samples within a matrix and within an individual oligosaccharide, yet allows oligosaccharides of widely different raw peak areas to be compared on a similar graphical scale. The direct comparisons of these scaled intensities between sample matrices or between oligosaccharides, however, cannot be made.

Targeted analyses, “Absolute Quantitation” data are reported in mg/L units. For statistical analysis, missing values were imputed with the limit of detection for that assay.

Significant differences between feeding groups (FF vs. BF) and between secretor and nonsecretor blood groups (BF Se- vs. BF Se+) were assessed by Mann-Whitney U test. Differences were considered significant if p value <0.05. Pearson Correlations were used to assess the degree of association of HMO concentrations in breast milk with those in the corresponding infant's plasma or urine. Associations were performed on imputed, log-transformed data, within similar data type, log (peak area) or log (mg/L). Correlations were considered significant if p value <0.05.

Data reduction was performed using Microsoft Excel 2010 and GraphPad Prism 5. Descriptive statistics are given as means ± standard error of the mean (SEM).

## Results

**Figure 2:**
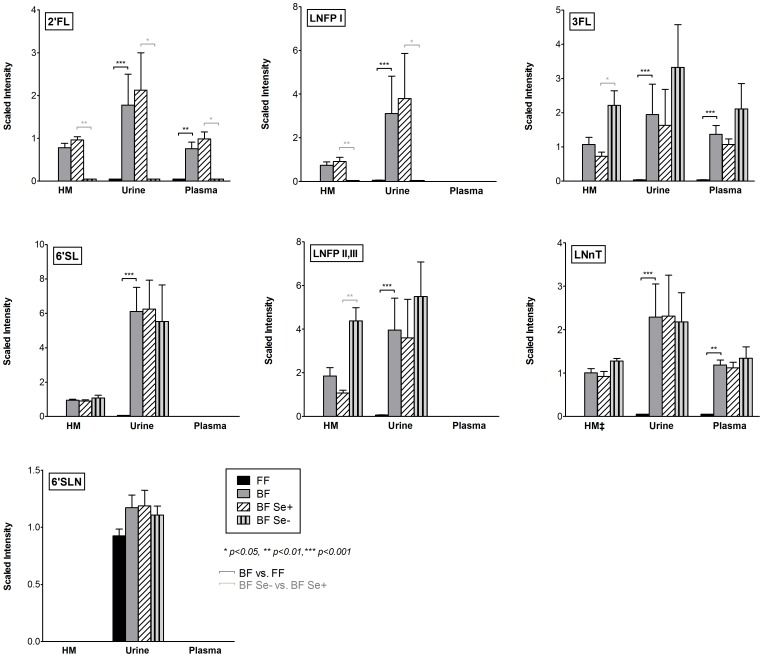
Relative quantitation of HMOs. Bar plots indicating the relative scaled intensities and Standard error of the mean (SEM) grouped according to feed. (FF vs. BF) and secretor status (BF Se+ vs. BF Se-); (for n, see Table 1). ‡LNnT measurement for breast milk (HM) as LNT, LNnT Isobar; Significant differences were assessed by Mann-Whitney *U* test where *p<0.05, **p<0.01, and ***p<0.001. Quantitative comparisons between sample matrices and between oligosaccharides cannot be made; quantitative comparisons between samples within a matrix and within individual oligosaccharides are valid.

Global untargeted profiling revealed the presence of seven milk oligosaccharides (2′FL, 3FL, LNnT, LNFP I, LNFP II,III, 6′SL and 6′SLN) in the infant urine samples (Figure 2), and all except 6′SLN were also present in breast milk. With the exception of 6′SLN, milk glycans were uniquely present in the urine of breastfed infants and not in those fed formula. We also found measurable amounts of intact 2′FL, 3FL and LNnT in the plasma of breastfed infants.

We classified infant-mother dyads as secretors (13/17, 76%) and nonsecretors (4/17, 24%) according to presence of 2′FL in the mother's breast milk. Using the 2′FL/3FL ratio for secretor determination (Table 1), we identified an additional subject as a potential nonsecretor (5/17, 29%). Figure 2 shows the relative quantitative patterns and relative levels for breast milk, urine and plasma, according to feeding group (FF vs. BF) and secretor status (BF *Se*- vs. BF *Se*+). Relative levels can only be compared within sample matrices and within individual HMOs, not across matrices or oligosaccharides. Milk samples from the four nonsecretors exhibited no LNFP I, 4-fold higher levels of LNFP II,III (p<0.01), and nearly 3-fold higher levels of 3FL (p<0.05) compared to secretors. As expected, the presence of 2′FL and LNFP I was found only in the infants of secretor mothers. As with the breast milk, levels of 3FL were higher in the plasma and urine of the nonsecretors compared to secretors; however the difference did not reach significance. There were no differences in relative quantitative levels of 6′SL or LNnT between secretors and nonsecretors in breast milk, urine or plasma (6′SL not detected in plasma).

**Table 1:** 2′FL/3FL ratios for breast milk.

		Raw Peak Area			
	Se status by 2′FL^a^	3FL	2′FL	2′FL/3FL	Se status by 2′FL/3FL^b^
HM 1	Se-	79670	*<167743*	2.1	Se-
HM 2	Se-	33898	*<167743*	4.9	Se-
HM 3	Se-	68712	*<167743*	2.4	Se-
HM 4	Se-	97276	*<167743*	1.7	Se-
**HM 5**	**Se+**	**48451**	**167743**	**3.5**	**Se-**
HM 6	Se+	28054	730711	26.0	Se+
HM 7	Se+	16163	808438	50.0	Se+
HM 8	Se+	11693	813825	69.6	Se+
HM 9	Se+	17198	826190	48.0	Se+
HM 10	Se+	*<6806*	898614	132.0	Se+
HM 11	Se+	6806	904175	132.8	Se+
HM 12	Se+	41078	923871	22.5	Se+
HM 13	Se+	29337	963326	32.8	Se+
HM 14	Se+	12967	1012804	78.1	Se+
HM 15	Se+	11872	1027943	86.6	Se+
HM 16	Se+	33751	1079979	32.0	Se+
HM 17	Se+	22922	1157248	50.5	Se+
Total # Se-	4				5
Total # Se+	13				12
%Se-	24%				29%

Relative quantitative data as raw peak areas.

a Presence of 2″FL indicates secretor.

b 2″FL/3FL ratio of >6.5 indicates secretor.

**Figure 3:**
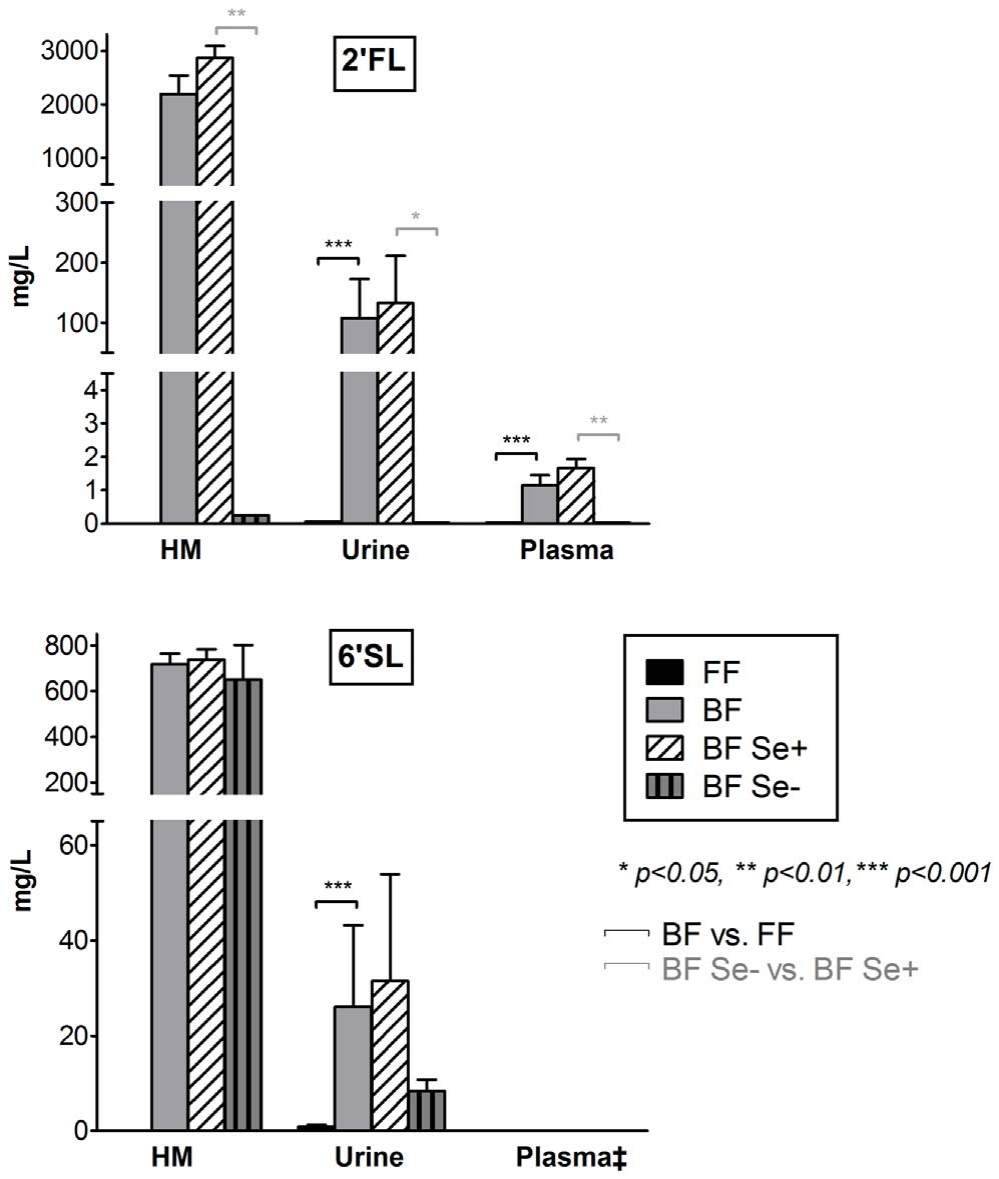
Absolute quantitation of HMOs. Bar plots indicating mean concentrations (mg/L) and Standard error of the mean (SEM) of 2′FL and 6′SL grouped according to feed (FF vs. BF) and secretor status (BF Se+ vs. BF Se-); (for n, see Table 1). ‡6′SL not detected in any plasma samples in the Global analysis. Significant differences were assessed by Mann-Whitney U test where *p<0.05, **p<0.01, and ***p<0.001.

The absolute concentration of 2′FL in the secretors' milk ranged from 649–3880 mg/L (mean 2866 mg/L) and was below the detection limit for the nonsecretors' milk (Table 2 and Figure 3). The milk level of 6′SL ranged from 288–982 mg/L (mean 718 mg/L). As with the relative quantitation, the targeted analysis revealed no differences in milk levels of 6′SL between secretors and nonsecretors.

**Table 2:** 2′FL and 6′SL Concentrations According to Feed and Secretor Status and Relative to Breast Milk Concentration.

		Mean ± SEM (mg/L)					P value	
		FF	BF	BF Se+	BF Se-	Relative Fraction*	BF vs FF	BF Se- vs BF Se+
**2′FL**	Urine	<0.06	108±65	133±78	<0.03	**4.0%**	**<0.001**	**<0.05**
	n =	17	16	13	3			
	Plasma	<0.03	1.16±0.29	1.66±0.27	<0.03	0.1%	**<0.001**	**<0.01**
	n =	14	13	9	4			
	HM	NA	2192±347	2866±222	<0.25			**<0.01**
	n =		17	13	4			
**6′SL**	Urine	0.9±0.3	26.1±17.2	31.5±22.4	8.3±2.4	**4.0%**	**<0.001**	NS
	n =	18	17	13	4			
	Plasma	ND	ND					
	n =	14	14					
	HM	NA	718±47	739±44	651±151			NS
	n =		17	13	4			

Absolute quantitative data (mg/L).

ND: not detected (6′SL not detected in any plasma samples in the Global analysis); NA: not applicable; NS: not significant; SEM: standard error of the mean.

*Concentration relative to breast milk level; BF Se+ group only for 2′FL, BF group for 6′SL.

Urine levels of both 2′FL (133±78 mg/L for BF *Se*+) and 6′SL (26.1±17.2 mg/L for BF and 0.9±0.3 mg/L for FF) showed significant differences between breastfed and formula-fed infants (p<0.001). There were significant differences between secretors and nonsecretors for 2′FL (p<0.05), but similar to the breast milk, there were no significant differences in urine levels of 6′SL between secretors and nonsecretors.

Concentrations of 2′FL in plasma (1.66±0.27 mg/L for BF *Se*+) showed significant differences between breastfed and formula-fed infants (p<0.001), and between secretors and nonsecretors (p<0.01) (Table 2 and Figure 3). 6′SL was not detected in any of our plasma samples.

**Figure 4:**
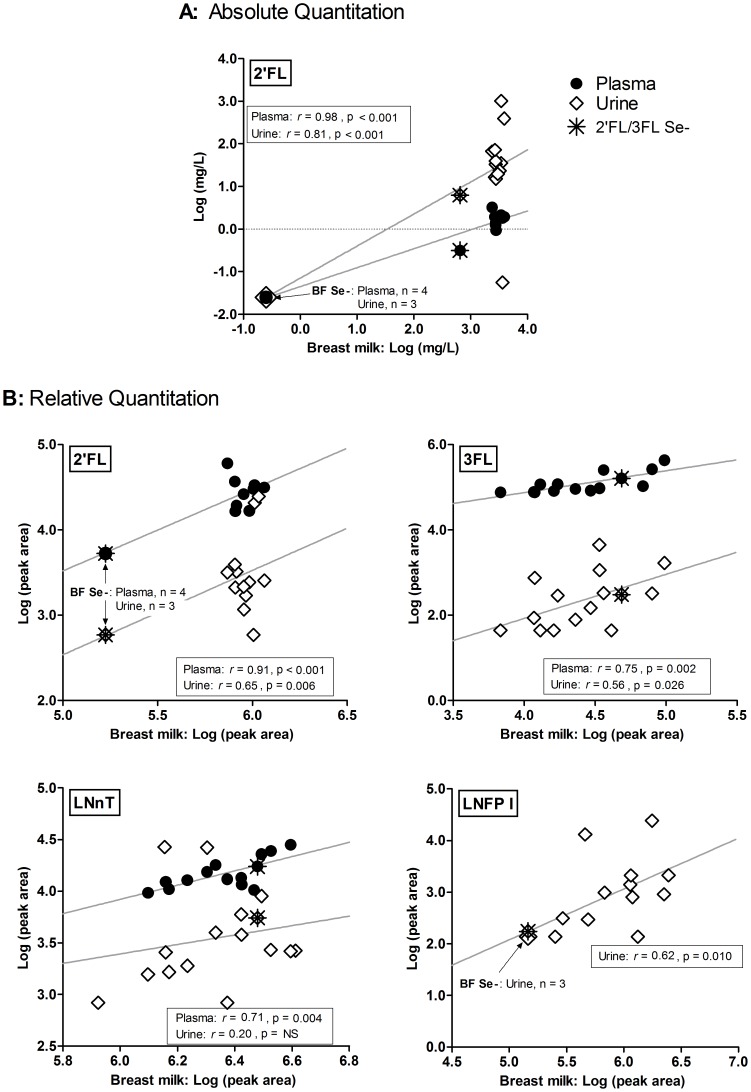
Correlation of infant plasma and urine HMO levels with breast milk concentrations. Scatter plots showing the association between individual HMO levels in breast milk and those in the corresponding infant plasma or urine.Pearson correlation statistics of log transformed data are inset. A: Absolute quantitation of 2′FL [Log (mg/L)]; B: Relative quantitation of 2′FL, 3FL, LNnT and LNFP I [Log (peak area)]. Solid lines represent best fit through the data points; (for n, see Table 2). (Plasma: black circles, Urine: white diamonds, Nonsecretor subject by 2′FL/3FL ratio: asterisk). NS, not significant.

Levels of several HMOs in urine and plasma corresponded with levels of those ingested (Table 3 and Figure 4). Pearson's correlation analyses of absolute quantitative data [Log (mg/L)] (Figure 4A) showed significant positive correlations for concentration of 2′FL in breast milk with both those in plasma (*r* = 0.98, 95% CI: 0.93 to 0.99, p<0.001), and with those in urine (*r* = 0.81, 95% CI: 0.53 to 0.93, p<0.001). Concentration of 6′SL in urine did not show correlation. Using relative quantitation [Log (peak area)] (Figure 4B), plasma values of 2′FL, 3FL and LNnT all showed positive correlations with breast milk (*r* = 0.91, 95% CI: 0.74 to 0.97, p<0.001; *r* = 0.75, 95% CI: 0.37 to 0.92, p = 0.002; and *r* = 0.71, 95% CI: 0.29 to 0.90, p = 0.004 respectively). 2′FL, 3FL and LNFP I urine levels also showed positive correlations with breast milk (*r* = 0.65, 95% CI: 0.23 to 0.87, p = 0.006; *r* = 0.56, 95% CI: 0.08 to 0.82, p = 0.026; and *r* = 0.62, 95% CI: 0.18 to 0.85, p = 0.010 respectively). While levels detected were clear evidence of intact absorption, relative fractions of the amount ingested appear to be low, 0.1% for plasma and 4% for urine (Table 2).

**Table 3:** HMO Urine and Plasma Level Correlations to Breast Milk Concentrations.

				Pearson Correlation^a^		
		Breast Milk compared to Infant:	Number of XY Pairs	r	P value	95% CI
**Absolute Quantitation**	**2′FL**	Urine	16	**0.81**	**<0.001**	0.53 to 0.93
		Plasma	13	**0.98**	**<0.001**	0.93 to 0.99
	**6′SL**	Urine	17	0.09	NS	−0.41 to 0.55
		Plasma	NT			
**Relative Quantitation**	**2′FL**	Urine	16	**0.65**	**0.006**	0.23 to 0.87
		Plasma	14	**0.91**	**<0.001**	0.74 to 0.97
	**3FL**	Urine	16	**0.56**	**0.026**	0.08 to 0.82
		Plasma	14	**0.75**	**0.002**	0.37 to 0.92
	**LNnT**	Urine	16	0.20	NS	−0.33 to 0.63
		Plasma	14	**0.71**	**0.004**	0.29 to 0.90
	**LNFP I**	Urine	16	**0.62**	**0.010**	0.18 to 0.85
		Plasma	14	ND		
	**LNFP II,III**	Urine	16	0.41	NS	−0.10 to 0.75
		Plasma	14	ND		
	**6′SL**	Urine	16	0.07	NS	−0.44 to 0.55
		Plasma	14	ND		
	**6′SLN**	Urine	16	NC		
		Plasma	14	ND		

a Pearson Correlations performed on imputed, log-transformed data, within similar data type, log (peak area) or log (mg/L).

NS, not significant; ND, not detected; NT, not tested; NC, not calculable.

CI, confidence interval; P value (two-tailed).

## Discussion

Here, we show measurable (1–133 mg/L) amounts of human milk oligosaccharides in the plasma and urine of breastfed but not formula-fed infants, and show significant correlations with those levels found in the ingested milk. While it is well-know that levels of HMOs vary between individuals and over the course of lactation, Thurl et al. [6] found 2′FL, 3FL and LNnT to vary little between days 14, 30 and 60 postpartum (2′FL - 3040, 2960, and 2820 mg/L respectively, not different; 3FL - 380, 420, 560 mg/L respectively, 15 and 30 days not different; LNnT - 280, 230, 230 mg/L respectively, not different). As such, the different collection time points used in our study should have little impact on the levels of these HMOs in our breast milk samples, and thus the ability to establish correlations between those levels in breast milk and in plasma.

The frequency of secretors in our study was 76%, in the range reported for African and European populations (60–80% secretors) [7], [8]. Using the 2′FL/3FL ratio to predict secretor status, only one of the samples analyzed in this study would be assigned nonsecretor status even though it contains minute amounts of 2′FL. However, this sample would probably be assigned secretor based on the ability of *Ulex Europaeus* agglutinin I to bind to its glycoproteins. Breast milk levels of 2′FL and 6′SL, as well as fold differences of 3FL and LNFP II between nonsecretors and secretors were also consistent with ranges previously reported (2′FL 2590–4570 mg/L, 6′SL 490–1770 mg/L; Se−/Se+: LNFP II,III 3x [1630/600], 3FL 4x [1790/420]) [6].

Of the 20 major HMOs, our untargeted analysis revealed detectable quantities of seven HMOs (2′FL, 3FL, LNnT, LNFP I, LNFP II,III, 6′SL and 6′SLN; LNFPII and LNFPIII are reported as an isobar as these molecules have the same molecular weight and formula, and fragmentation does not distinguish the two isoforms) in urine, all except 6′SLN were present in breast milk, and 2′FL, 3FL and LNnT were also in plasma.

In a recent study of 55 mother-infant dyads, 2′FL was identified in infant plasma by HPAEC and LC/MS/MS in the same concentration range (0–2.25 mg/L) as determined in our analyses (3FL was not tested). They also found similar levels of HMOs in breast milk, 6′SL (29.3–726 mg/L), 2′FL (0–3.8 g/L) and 3FL (0.04–1.10 g/L, our data relative quantitation only). Unlike our findings, however, they identified both 3′SL (0.10–0.78 mg/L) and 6′SL (0.05–0.68 mg/L) in plasma, and 3′SL (54.3–225 mg/L,) in breast milk [37]. These disparities could be due to differences in biochemical extraction methodologies and/or detection processes of HMO analyses. We hypothesize that many additional HMOs could be identified in urine and plasma with the development of more sensitive and specific targeted analyses.

We did not find 6′SLN in breast milk or plasma, we did however, find it in urine from both breastfed and formula-fed infants. Similarly, De Leoz et al. [38] detected 6′SLN in urine and feces of breastfed infants, but not in mother's milk. 6′SLN has also been identified in bovine milk, which may account for its presence in the urine of the formula-fed infants [39]. Rudloff et al. [30] suggested the presence of ‘modified’ HMOs in urine that may come directly from breast milk itself, or that may be synthesized endogenously from smaller precursors. It may also be a breakdown product resulting from bacterial fermentation of glycoconjugates in milk or the shedding of Neu5Acα2-6Galβ1-4GlcNAc structures from cell surfaces [40], [41].

Prieto [42] demonstrated that fructooligosaccharides (FOS), which are carbohydrates added to infant formulas to emulate HMO, can be detected in the urine of infants fed formulas supplemented with these carbohydrates. This was possible because FOS generate well defined signals corroborated by co-elution with well characterized standards. The infant formula used as a control in this study contained galactooligosaccharides (GOS) which are also added to infant formulas in an effort to functionally mimic HMO [43]. GOS are heterogeneous mixtures of isomers and chain lengths and we did not have the methodology to identify structures in urine or plasma that could be conclusively attributed to the ingestion of GOS by formula fed infants. Additionally, we did not identify major MW signals that would suggest that GOS were detected in our analyses.

Neutral HMOs have been shown to be transported across the intestinal epithelium by receptor-mediated transcytosis as well as via paracellular pathways, and have been measured in the intracellular compartment. Acidic glycans seem to be translocated only via paracellular flux [31]. Eiwegger showed a 4–14% uptake of neutral HMOs across the intestinal epithelium [44]. Milk oligosaccharides have also been hypothesized to be taken up directly from the gut lumen by dendritic cells [45]. In addition to demonstrating the intestinal absorption of HMOs and the likely mechanisms involved, these data indicate that at least some milk glycans are available at the intracellular level where they have the potential to directly modulate cell signaling.

Similar to our data, several studies in exclusively breastfed infants found urinary HMO profiles resembling those of the mothers' milk and a renal excretion of 1–3% of individual HMOs. Several of these studies used a bolus of ^13^C-labelled galactose fed to lactating women and showed the ^13^C-Gal to be directly incorporated into the mothers' breast milk oligosaccharides. These ^13^C-HMOs were then detected in the infants' urine, reaching peak levels at 8–12 hours after the mothers were fed, and were still detectable 36 hours later. This suggests that HMOs from mother's milk may stay in infant's circulation for several hours after absorption before being excreted via urine. Additionally, variations in retention time in the infant suggest that neutral and acidic HMOs may be utilized differentially [30], [46]–[48].

There is increasing evidence that milk glycans provide specific benefits to the immune system and neuronal development of the breastfed infant [49]. In order to exert systemic effects however, HMOs must be absorbed and transported in the peripheral blood to specific cells where they might interact. *In vitro* studies suggest that milk glycans can directly modulate immune cell responses at very low concentrations. HMOs at concentrations of 1 µg/mL have been shown to enhance the maturation of Th1 lymphocyte responses, as evidenced by the induction of IFN-γ and IL-10 but not IL-4 in cord blood mononuclear cells [44], [50]. Low concentrations of HMOs (12.5 µg/mL) exhibit anti-inflammatory properties by inhibiting leukocyte rolling and adhesion, modulating signaling pathways, and dampening platelet-neutrophil mediated inflammation [51], [52]. Doses of 2′FL as low as 5 µg/mL (significant at 30 µg/mL) decrease cell proliferation and reduce the production of IL-12 and IFN-γ, while increasing IL-10 in LPS-stimulated adult human mononuclear cells [53]. These immunomodulatory effects may be mediated by signaling through specific glycan-lectin receptors on immune cells [21], [29], [51], [53], [54].

In conclusion, at a minimum, some small molecular weight HMOs (2′FL, 3FL, LNnT) are absorbed intact into the circulation and excreted in the urine without metabolic modification at levels that correlate with their dietary intake from breast milk. Our findings are consistent with previous observations that described the presence of human milk oligosaccharides in the urine of breastfed infants, but we show for the first time measurable amounts of HMOs are also present in the plasma and at levels relative to the amount fed. Our data show that systemic effects of milk glycans via direct interaction with the immune system outside of the gastrointestinal tract are possible, and that the levels shown to have biological effects *in vitro* are physiologically achievable.
